# Determinants of COVID-19 vaccine readiness and hesitancy among adults in sub-Saharan Africa

**DOI:** 10.1371/journal.pgph.0000713

**Published:** 2023-07-14

**Authors:** Sulemana Watara Abubakari, Firehiwot Workneh, Kwaku Poku Asante, Elena C. Hemler, Isabel Madzorera, Dongqing Wang, Abbas Ismail, Nega Assefa, Temesgen Azemraw, Bruno Lankoande, Abdul Razak Nuhu, Angela Chukwu, Frank Mapendo, Ourohiré Millogo, Adedokun A. Olufemi, Daniel Okpara, Valentin Boudo, Mary Mwanyika-Sando, Yemane Berhane, Till Baernighausen, Ayoade Oduola, Said Vuai, Ali Sie, Abdramane Soura, Japhet Killewo, Raji Tajudeen, Wafaie W. Fawzi, Emily R. Smith

**Affiliations:** 1 Research and Development Division, Kintampo Health Research Centre, Ghana Health Service, Kintampo North Municipality, Bono East Region, Ghana; 2 Department of Epidemiology and Biostatistics, Addis Continental Institute of Public Health, Addis Ababa, Ethiopia; 3 Department of Global Health and Population, Harvard T.H. Chan School of Public Health, Harvard University, Boston, Massachusetts, United States of America; 4 College of Natural and Mathematical Sciences, University of Dodoma, Dodoma, Tanzania; 5 College of Health and Medical Sciences, Haramaya University, Harar, Ethiopia; 6 Institut Superieur des Sciences de la Population, University of Ouagadougou, Ouagadougou, Burkina Faso; 7 Department of Statistics, University of Ibadan, Ibadan, Nigeria; 8 Africa Academy for Public Health, Dar es Salaam, Tanzania; 9 Nouna Health Research Center, Nouna, Burkina Faso; 10 University of Ibadan Research Foundation, University of Ibadan, Ibadan, Nigeria; 11 Heidelberg Institute of Global Health, University of Heidelberg, Heidelberg, Germany; 12 Africa Health Research Institute, KwaZulu-Natal, South Africa; 13 Department of Epidemiology and Biostatistics, Muhimbili University of Health and Allied Sciences, Dar es Salaam, Tanzania; 14 Division of Public Health Institutes and Research, Africa Centres for Disease Control and Prevention, Addis Ababa, Ethiopia; 15 Department of Nutrition, Harvard T.H. Chan School of Public Health, Harvard University, Boston, Massachusetts, United States of America; 16 Department of Epidemiology, Harvard T.H. Chan School of Public Health, Harvard University, Boston, Massachusetts, United States of America; 17 Department of Global Health, Milken Institute School of Public Health, George Washington University, Washington, DC, United States of America; 18 Department of Exercise and Nutrition Sciences, Milken Institute School of Public Health, George Washington University, Washington, DC, United States of America; University of Oslo Faculty of Medicine: Universitetet i Oslo Det medisinske fakultet, NORWAY

## Abstract

There is very limited data on the extent and determinants of COVID-19 vaccine hesitancy among adults living in sub-Saharan Africa since the global roll-out of vaccines began in 2021. This multi-country survey sought to investigate COVID-19 vaccine hesitancy and other predictors of readiness to get vaccinated. We conducted surveys among adults residing in nine urban and rural areas in Burkina Faso, Ethiopia, Ghana, Nigeria, and Tanzania in late 2021. Log binomial regression models were used to identify prevalence and factors associated with vaccine hesitancy and beliefs around COVID-19 misinformation. We completed a total of 2,833 interviews. Among all respondents, 9% had never heard of a COVID-19 vaccine, 12% had been vaccinated, and 20% knew someone else who had been vaccinated. The prevalence of vaccine hesitancy varied by country (Ethiopia 29%, Burkina Faso 33%, Nigeria 34%, Ghana 42%, Tanzania 65%), but not by rural or urban context. People who did not think the vaccine was safe or effective, or who were unsure about it, were more likely to be vaccine hesitant. Those who reported they did not have a trusted source of information about the vaccine (aPR: 1.25, 95% CI: 1.18,1.31) and those who thought the vaccine would not be made available to them within the year were more likely to be vaccine hesitant. Women were more likely to be vaccine hesitant (aPR: 1.31, 95% CI: 1.19,1.43) and believe COVID-19 falsehoods (aPR: 1.05, 95% CI: 1.02,1.08). The most commonly believed falsehoods were that the vaccine was developed too fast and that there was not enough information about whether the vaccine was effective or not. Educational campaigns targeted at misinformation and tailored to suit each country are recommended to build trust in COVID-19 vaccines and reduce hesitancy.

## Introduction

The World Health Organization (WHO) declared COVID-19 as a Public Health Emergency of International Concern (PHEIC) more than two years ago on January 30, 2020 [1]. As of March 2022, there were over 435 million reported infections and nearly 6 million reported deaths from COVID-19 [2]. At the 73rd World Health Assembly in May 2020, a resolution was passed recognizing the role of immunization as the key global public health strategy for preventing, containing, and minimizing transmission of COVID-19 [3]. The global fight against the COVID-19 pandemic cannot be won until there is a worldwide roll-out of vaccines that provide herd immunity and protect against severe morbidity and mortality [4]. Even as the SARS-CoV-2 infections become endemic, vaccination will likely remain key to preventing excess morbidity and mortality.

Discovery and approval of multiple safe and effective vaccines is an impressive accomplishment, but equitable access globally is critical to ending the COVID-19 pandemic. Accordingly, WHO is working with partners in the development, manufacturing, and deployment of safe and effective vaccines [5]. COVID-19 vaccine development has happened at an unprecedented pace to control the pandemic following WHO’s recommendation and currently there are 10 vaccines authorized for emergency use listing, 197 vaccine candidates for clinical trials, and 153 vaccines pre-clinical trials ongoing globally as at April 29^th^ 2022 [6]. Almost all of these vaccine developments are being undertaken in high-income countries. However, the rapid pace of COVID-19 vaccine development has become one of the primary reasons for vaccine hesitancy [7].

Vaccine hesitancy is the reluctance or delay in acceptance or refusal of vaccination despite the availability of safe, recommended, and available vaccines [8]. Vaccine hesitancy was a growing concern prior to COVID-19, and in 2019, WHO identified it as one of the ten main threats to global health [9]. Different factors have been identified as influencing COVID-19 vaccine hesitancy including gender [10], knowledge of COVID-19 vaccines [11], safety and importance of the vaccines [12], and mistrust in sources conveying information about the vaccines [10,13]. Vaccine hesitancy is a result of increased concerns from individuals and communities questioning the safety of vaccines, seeking alternative vaccination schedules and delaying or refusing vaccination [13,14]. For instance, an analysis of three years of WHO/United Nations Children Fund (UNICEF) Joint Report Form (JRF) data (2015–2017) showed that vaccine hesitancy was prevalent in over 90% of the 194 countries across all WHO regions and all categories of country income levels [9,15]. More recent outbreaks of measles, a vaccine-preventable disease in the United States of America (USA) and Europe, are largely attributable to vaccine hesitancy [16–18]. The 2003–2004 polio vaccine boycott in Nigeria [14,19] is another example of growing vaccine hesitancy on a global scale.

As the roll-out of COVID-19 vaccines gained momentum across the world, there were increasing tensions due to supply-related challenges, rising trends of vaccine nationalism, and inequitable vaccine access especially in poor-resource countries in sub-Saharan Africa (SSA) [20,21]. Data on vaccine hesitancy from low- and middle-income countries (LMICs), especially from SSA, have been limited [7], and COVID-19 vaccine hesitancy is an additional concern [22,23]. As LMICs are starting to receive larger quantities of COVID-19 vaccines [4], it is important to understand the determinants of vaccine acceptability with the intention of creating strategies for increasing vaccine coverage in order to rapidly bring the pandemic to an end [10]. Promoting the uptake of vaccines requires understanding whether people are willing to be vaccinated, the reasons why they are willing or unwilling to do so, and the most trusted sources of information in their decision-making [7].

In response to the COVID-19 public health emergency, the Africa Research Implementation Science and Education (ARISE) Network undertook a multi-country, rapid telephone survey to investigate COVID-19 vaccine risk perceptions and other predictors of vaccine readiness, building off of a baseline survey conducted in 2021 to study impacts of the COVID-19 pandemic. This survey aimed to understand current beliefs regarding COVID-19 vaccine hesitancy in order to develop strategies to promote the intention to get vaccinated against COVID-19 and to overcome hesitancy and refusal among households in SSA.

## Materials and methods

### Study areas and study population

This is a multi-country, repeated cross-sectional study conducted in five SSA countries, Burkina Faso, Ethiopia, Ghana, Nigeria, and Tanzania. Study areas were selected based on existing data collection infrastructure, previous experience working in study areas, research team capacity, and willingness to participate. Where possible, this survey used ongoing health and demographic surveillance systems or existing national surveys as sampling frames. First-round data was collected in 2020 in urban and rural areas in three countries; Burkina Faso, Ethiopia, and Nigeria. In this second round, two additional countries were added: Ghana (Kintampo) and Tanzania (Dodoma, Dar es Salaam). Details about each area in Burkina Faso, Ethiopia, and Nigeria were reported in detail previously [24]. Similar to the other countries, in both Tanzania and Ghana, Health and Demographic Surveillance System (HDSS) sites were used for sampling. In Tanzania the Dar es Salaam Urban Cohort Study (DUCS) and HDSS and Dodoma HDSS provided sampling frames. Similarly, Kintampo HDSS was used for sampling the households in Ghana. The urban study communities were in Ouagadougou in Burkina Faso, Addis Ababa in Ethiopia, Lagos in Nigeria, and Dar es Salaam in Tanzania. The rural communities were Nouna in Burkina Faso, Kersa in Ethiopia, Ibadan in Nigeria, Dodoma in Tanzania, and Kintampo in Ghana.

### Study design and sampling

This study used a novel mobile platform to collect survey data to assess predictors of vaccine hesitancy among adults. In each household, one adult aged 20 years or older was interviewed. In Burkina Faso, Ethiopia, and Nigeria, we re-contacted participants from the round 1 survey to conduct the survey for round 2. In order to compensate for the loss to follow up and reach the target sample size of 300 per site, we recruited new participants in each country using the same methods that were employed in the round 1 survey [24]. Participants with any working phone were included in this survey. During the actual data collection, both mobile and landline phones were used, but the majority of participants were contacted using their mobile phones. The interviews were conducted mainly during the day, but phone calls were also made at night and over the weekends to accommodate participants’ schedules.

### Data collection

All enumerators were trained on study procedures, including screening, consent, enrollment, and data collection, emphasizing confidentiality and safeguarding the participant’s rights and wellbeing. All interviews were conducted by trained enumerators in the local languages of each site using computer-assisted telephone interviewing (CATI). Research staff in each site conducted the interviews from virtual call centers. Verbal informed consent was obtained before the interview. Data were collected from July to December 2021 in all nine areas and the survey took 30–40 minutes. A standardized questionnaire developed in consultation with subject matter experts at participating institutions across the five countries was used for data collection. The practicability, validity, and interpretability of answers for the respective questions were confirmed by performing a pretest among adults in all survey areas. Based on the pre-test, slight modifications were made to the tool to refine it for each specific context. All questionnaires and participant materials were translated into the local languages of each area by experts familiar with the context in each site. The questionnaire assessed 1) sociodemographic information; 2) knowledge, practices, and perceptions of COVID-19; 3) mental health and COVID-19; and 4) knowledge, perceptions, beliefs, and hesitancy related to COVID-19 vaccines. The questionnaires are available at (https://africa.harvard.edu/covid-19-resources). During the phone interview, data collectors entered participant data into a mobile tablet-based data collection system, Open Data Kit. Data were uploaded to a secure server in each country after collection. A core data management team reviewed all data for completeness and quality using the same analysis codes and concatenated files from all study areas for pooled data analysis.

### Ethical approval

This study obtained ethical approval from the institutional review boards of Harvard T.H. Chan School of Public Health (Reference Number: IRB20-0909) and the ethical authorities in each country including: Nouna Health Research Center Ethical Committee Burkina Faso (Reference number: 2020-009-/MS/SG/INSP/ CRSN /CIE); National Ethics Committee Burkina Faso (Reference number: 2020-7-127); Institutional Ethical Review Board of Addis Continental Institute of Public Health Ethiopia (Ref. No.: ACIPH/IRB/005/2021); University of Ibadan Research Ethics Committee Nigeria (Ref. No. UI/SSHEC/2020/0017); Muhimbili University of Health and Allied Sciences Tanzania (Ref. No. DA 282/298/06/C/767); University of Dodoma Tanzania (Ref. No. MA.84/261/02/134); National Institute of Medical Research Tanzania (Ref. No. NIMR/HQ/R.8a/Vol. IX/3775); and Kintampo Health Research Centre Institutional Ethics Committee Ghana (Ref. No, KHRCIEC/2021-12).

### Inclusivity in global research

Additional information regarding the ethical, cultural, and scientific considerations specific to inclusivity in global research is included in the (S1 Checklist).

### Statistical analysis

Descriptive analyses were conducted by calculating means and standard deviations (SDs) for normally-distributed continuous variables, medians and interquartile ranges for skewed variables, and counts and percentages for categorical variables for each survey site. Respondents were classified as “vaccine hesitant” based on their response to the question about whether they would get the COVID-19 vaccine if it was available now. Those who responded: “No, would definitely not get it”; “Maybe, would wait and see what others do before getting it”; or “Unsure or undecided” were considered vaccine hesitant. Those who had already gotten vaccinated or said they “Yes, would definitely get it” were consider vaccine-ready or not-vaccine hesitant. Factors associated with vaccine hesitancy were identified using log-binomial or modified Poisson regression with robust variance models to generate adjusted prevalence ratios (aPR) with 95% confidence intervals (95% CIs). We preferred to sue this approach, since the traditional binary logistic regression overestimate the effect measures. Each model included demographic characteristics including country and rural status of residence, age, and sex as potential confounders. SAS version 9.4 was used for all analyses.

## Results

We completed a total of 2,830 interviews (S1 Fig), including 625 in Burkina Faso, 587 in Ethiopia, 663 in Nigeria, 654 in Tanzania, and 301 in Ghana. Just over half of all respondents were female, although only about one-third of the respondents were female in Addis Ababa, Ethiopia and Dodoma, Tanzania. The mean age of respondents was 42 years old (SD 12.5), with the youngest average age in Ethiopia and the oldest in Burkina Faso (Table 1).

**Table 1:** Sociodemographic characteristics of survey respondents from 9 study sites. (n = 2,830).

Characteristics	Burkina Faso	Burkina Faso	Ethiopia	Ethiopia	Nigeria	Nigeria	Tanzania	Tanzania	Ghana	Total
	Nouna	Ouaga	Addis	Kersa	Ibadan	Lagos	DSM	Dodoma	Kintampo	
**Female**	274 (84.6)	198 (65.8)	87 (30.1)	254 (30.1)	209 (56.0)	159 (54.8)	161 (52.4)	115 (33.1)	141 (46.8)	1598 (56.5)
**Respondent age Mean (SD)**	47.7 (12.9)	47.4 (10.1)	38.0 (12.2)	36.6 (9.4)	40.4 (13.1)	39.5 (11.8)	48.6 (12.5)	43.0 (10.1)	40.0 (13.6)	42.2 (12.5)
**Head of household**	244 (75.3)	238 (79.1)	211 (73.0)	265 (88.9)	187 (50.1)	155 (53.5)	224 (73.0)	184 (53.0)	161 (53.5)	1869 (66.0)
**Occupation**	** **									
Unemployed/Stay home parent	0 (0.0)	25 (8.3)	59 (20.4)	0 (0.0)	13 (3.5)	4 (1.4)	30 (9.8)	2 (0.6)	31 (10.3)	164 (5.8)
Student	2 (0.6)	2 (0.7)	2 (0.70)	9 (3.0)	59 (15.8)	26 (9.0)	1 (0.3)	0 (0.0)	11 (3.7)	112 (4.0)
Farmer	235 (72.5)	15 (5.0)	0 (0.0)	268 (89.9)	7 (1.9)	4 (1.4)	22 (7.2)	333 (96.0)	119 (39.5)	1003 (35.4)
Wage employment	13 (4.0)	35 (11.6)	60 (20.8)	15 (5.0)	100 (26.8)	103 (36.0)	49 (16.0)	4 (1.2)	46 (15.3)	425 (15.0)
Self-employed	31 (9.6)	131 (43.5)	107 (37.0)	10 (3.4)	129 (34.6)	117 (40.3)	172 (56.0)	6 (1.7)	118 (39.2)	821 (29.0)
Other	34 (10.5)	47 (15.6)	63 (21.8)	26 (8.7)	6 (1.6)	1 (0.3)	37 (12.1)	19 (5.5)	8 (2.7)	241 (8.5)
**Number of U5 children in household [Median (IQR)]**	1 (0,2)	1 (0,1)	0 (0,1)	1 (0,2)	0 (0,1)	0 (0,1)	0 (0,1)	1 (0,1)	1 (0,2)	1 (0,2)
**Highest level of education**	** **									
None, religious, literacy class	179 (55.3)	171 (56.8)	32 (11.1)	82 (27.5)	7 (1.9)	3 (1.0)	8 (2.6)	79 (22.8)	91 (30.2)	652 (23.0)
Some primary school education	75 (23.2)	50 (16.6)	51 (17.7)	125 (42.0)	13 (3.5)	2 (0.7)	7 (2.3)	33 (9.5)	53 (17.6)	409 (14.5)
Completed primary school	30 (9.3)	18 (6.0)	29 (10.0)	31 (10.4)	25 (6.7)	9 (3.1)	168 (54.7)	219 (63.1)	23 (7.6)	552 (19.5)
Some secondary/high school	23 (7.1)	42 (14.0)	48 (16.6)	30 (10.1)	26 (7.0)	9 (3.1)	43 (14.01	10 (2.9)	43 (14.30)	274 (9.7)
Completed secondary/high school	4 (1.2)	6 (2.0)	58 (20.1)	11 (3.70	51 (13.7)	39 (13.5)	49 (16.0)	6 (1.7)	57 (18.9)	281 (9.9)
Tertiary education or higher	9 (2.8)	11 (3.7)	71 (24.6)	19 (6.4)	239 (64.1)	215 (74.1)	30 (9.8)	0 (0.0)	34 (11.3)	628 (22.2)

**Fig 1:**
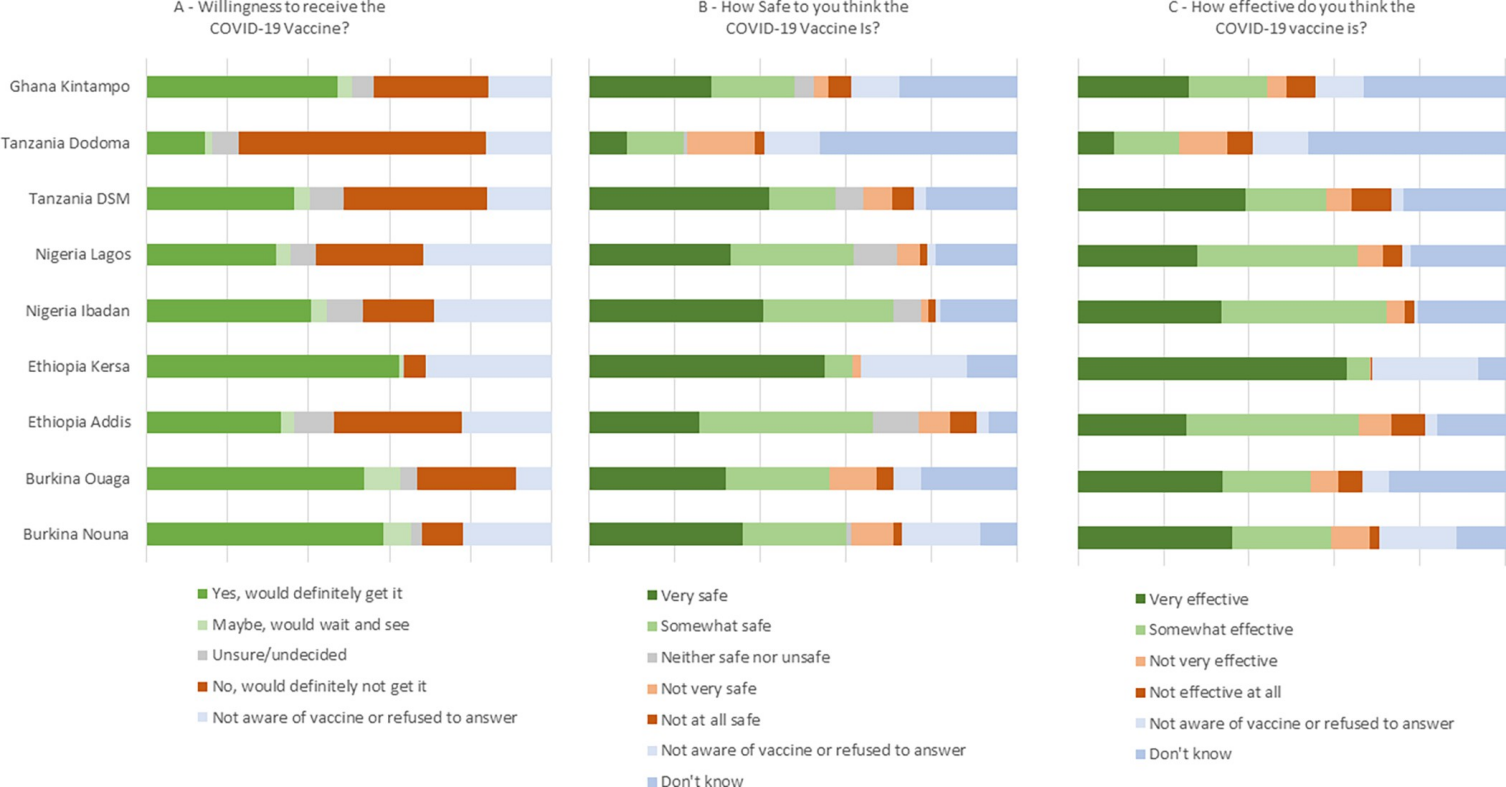
Willingness to receive (A), perceived effectiveness (B), and perceived safety (C) of the COVID-19 vaccine by country and overall.

Among all respondents, 9% (n = 258) had never heard of a COVID-19 vaccine, 12% had been vaccinated (n = 344), and 20% knew someone else who had been vaccinated (n = 575). The proportion of respondents who said they definitely would receive the vaccine was highest in Kersa Ethiopia (62%), followed by Nouna Burkina Faso (58%), Ouagadougou Burkina Faso (54%), and Kintampo Ghana (47%). Respondents in Dodoma, Tanzania were least likely to say they would definitely get the vaccine (14%) and most likely to say they definitely would not get it (61%) (Fig 1A). Respondents in Kersa Ethiopia were the most likely to think the vaccine was very or somewhat effective and very or somewhat safe (Fig 1B and 1C).

The most common reason respondents said they would get the vaccine was to keep themselves and their families safe. More than half of the respondents in Nouna, Ouagadougou, Addis Ababa, Ibadan, and Kintampo said they would get the vaccine if their doctor suggested it, though only about one-third of respondents in Tanzania report that as a reason they would get the vaccine (Table 2). In most study areas, the most common reason respondents said they would not get the vaccine is because they do not think it is needed (Table 2). In Addis Ababa and Kintampo, concern about side effects ranked as the most commonly reported reason for not getting the vaccine. Overall, less than 5% of the respondents mentioned misinformation-related reasons for not wanting to get the vaccine such as fear of getting illness or autism from the vaccine, concerns regarding infertility, fear of microchipping or new world order. However, there was variation across countries. For example, concerns about the vaccine and fertility were more common (7%) in Ouagadougou and Kintampo and fear of microchipping were more common in Ibadan and Kintampo (9–10%). Concerns about eligibility or safety due to pregnancy or breastfeeding also came up as other reasons people would not get the vaccine.

**Table 2:** Reported reasons for vaccine readiness or vaccine hesitancy by country.

Characteristics	Burkina Faso	Burkina Faso	Ethiopia	Ethiopia	Nigeria	Nigeria	Tanzania	Tanzania	Ghana	Total
	Nouna	Ouaga	Addis	Kersa	Ibadan	Lagos	DSM	Dodoma	Kintampo	
**Reasons you would get the vaccine** ^**1**^	N = 226	N = 203	N = 198	N = 200	N = 300	N = 201	N = 189	N = 89	N = 182	N = 1788
I would get the vaccine to keep myself safe	204 (90.3)	193 (95.1)	187 (94.4)	194 (97.0)	265 (88.0)	186 (92.5)	147 (77.8)	66 (74.2)	173 (95.1)	1615 (90.3)
I would get the vaccine to keep my family safe	205 (90.7)	1989(93.1)	185 (93.4)	158 (79.0)	264 (88.0)	185 (92.0)	146 (77.3)	58 (65.2)	167 (91.8)	1557 (87.1)
I would get the vaccine because my friends/family suggested I get it	126 (55.8)	136 (67.0)	101 (51.0)	77 (38.5)	175 (58.3)	73 (36.3)	58 (30.7)	10 (11.2)	119 (65.4)	875 (48.9)
I would get the vaccine because my doctor suggested I get it	171 (75.7)	191 (94.1)	114 (57.6)	80 (40.0)	186 (62.0)	96 (47.8)	66 (34.9)	22 (24.7)	160 (87.9)	1086 (60.7)
**Reasons you would not get the vaccine** ^**2**^	N = 64	N = 110	N = 126	N = 19	N = 103	N = 102	N = 146	N = 221	N = 98	N = 989
Do not think it is needed	32 (49.2)	61 (52.6)	37 (28.7)	6 (31.6)	47 (40.9)	38 (34.6)	42 (28.8)	168 (69.4)	17 (15.2)	448(42.5)
Do not think I am at risk of getting COVID	19 (29.2)	24 (20.7)	19 (14.7)	6 (31.6)	18 (15.7)	12 (10.9)	21 (14.4)	16 (6.6)	10 (8.9)	145 (13.8)
Do not think the vaccine is effective against COVID-19	11 (16.9)	14 (12.1)	48 (37.2)	1 (5.3)	23 (20.0)	22 (20.0)	17 (11.6)	20 (8.3)	6 (5.4)	162 (15.4)
Heard/read negative media reports	4 (6.2)	20 (17.2)	24 (18.6)	2 (10.5)	30 (26.1)	25 (22.7)	15 (10.3)	11 (4.6)	7 (6.3)	138(13.1)
Do not think the vaccine is safe/ It was developed too fast	8 (12.3)	19 (16.4)	22 (17.1)	2 (10.5)	14 (12.2)	21 (19.1)	5 (3.4)	7 (2.9)	9 (8.0)	107 (10.2)
Concerned about side effects	14 (21.5)	44 (37.9)	56 (43.4)	2 (10.5)	19 (16.5)	19 (17.3)	1 (0.7)	15 (6.2)	20 (17.9)	190 (18.0 )
Fear of getting an experimental vaccine or worse quality vaccine	7 (10.8)	5 (4.3)	3 (2.3)	0 (0)	8 (7.0)	10 (9.1)	0 (0)	2 (0.8)	1 (0.9)	36 (3.4 )
Fear getting COVID-19 disease from the vaccine	14 (21.5)	7 (6.0)	11 (8.5)	4 (21.1)	7 (6.1)	20 (18.2)	1 (0.7)	4 (1.7)	20 (17.9)	88 (8.4)
Fear getting other illnesses / autism from the vaccine	17 (26.2)	3 (2.6)	1 (0.8)	0 (0)	6 (5.2)	10 (9.1)	2 (1.4)	5 (2.1)	7 (6.3)	51 (4.8)
Fear the vaccine will cause infertility / sterilization / population control	3 (4.6)	16 (13.8)	6 (4.7)	1 (5.3)	3 (2.6)	5 (4.6)	4 (2.7)	4 (1.7)	8 (7.1)	50 (4.7)
Religious reasons/church or religion advises against	0 (0)	1 (0.9)	15 (11.6)	0 (0)	2 (1.7)	2 (1.8)	2 (1.4)	12 (5.0)	1 (0.9)	35 (3.3)
Fear of microchipping	0 (0)	15 (12.9)	3 (2.3)	0 (0)	11 (9.66)	3 (2.7)	0 (0)	0 (0)	10 (8.9)	42 (3.98)
Fear of New World Order	0 (0)	0 (0)	1 (0.8)	0 (0)	8 (7.0)	2 (1.8)	0 (0)	1 (0.4)	3 (2.7)	15 (1.4)
Had a bad experience or reaction with previous vaccinations	0 (0)	5 (4.3)	2 (1.6)	0 (0)	1 (0.9)	1 (0.9)	0 (0)	2 (0.8)	3 (2.7)	14 (1.3)
Concerned because I have a chronic condition e.g. diabetes, hypertension and not sure it is safe for people with my condition	4 (6.2)	1 (0.9)	2 (1.6)	0 (0)	2 (1.7)	1 (0.9)	7 (4.8)	1 (0.4)	1 (0.9)	19 (1.8)
Personal liberty / do not want bodily intrusion	3 (4.6)	7 (6.0)	7 (5.4)	0 (0)	2 (1.7)	1 (0.9)	3 (2.1)	12 (5.0)	1 (0.9)	36 (3.4)

^1^ Among n = 1788 respondents who already got the vaccine or said yes, maybe, or unsure to the question "How much do you agree with this statement? If a vaccine for COVID-19 were available now, I would definitely get it".

^2^ Among n = 989 respondents who said no, maybe, unsure, or refused to answer the question "How much do you agree with this statement? If a vaccine for COVID-19 were available now, I would definitely get it".

More than one in five respondents were concerned that the vaccination site would be far from home or inconvenient to get to, that they wouldn’t be able to afford the vaccine, that they would not be prioritized as quickly as the wealthy or elite, or than they needed their husband or family’s consent to get the vaccine; these concerns varied by study area (Table 3). Across all study areas, healthcare workers were the most likely to influence opinions about whether to get the vaccine (overall 57%, with range from 30% in Dar es Salaam to 74% in Ouagadougou). About one third of respondents said that family or loved ones and religious leaders would also influence their decision. Similarly, respondents cited traditional media sources (television, radio, or newspaper), medical professionals, and government communications as the places they looked for trusted news. Social media and the internet were least commonly noted as a trusted source of information, although about half of respondents in Nigeria said they turned to these sources for information. When asked about when they thought a COVID-19 vaccine would be made available to them, about one-fifth of respondents said never and more than one-third said they didn’t know (Table 3).

**Table 3:** Perceived barriers and enablers to getting vaccinated by study site.

Characteristics	Burkina Faso	Burkina Faso	Ethiopia	Ethiopia	Nigeria	Nigeria	Tanzania	Tanzania	Ghana	Total
	Nouna	Ouaga	Addis	Kersa	Ibadan	Lagos	DSM	Dodoma	Kintampo	
**Barriers to getting the COVID-19 vaccine, if it was available?**										
	n = 324	n = 301	n = 289	n = 298	n = 373	n = 290	n = 307	n = 347	n = 301	n = 2830
The location of vaccination sites is far	112 (34.57)	88 (29.24)	33 (11.42)	147 (49.33)	68 (18.23)	111 (38.28)	34 (11.07)	12 (3.46)	83 (27.57)	688 (24.31)
I don’t want to / cannot miss work	36 (11.11)	54 (17.94)	91 (31.49)	14 (4.7)	51 (13.67)	105 (36.21)	25 (8.14)	4 (1.15)	60 (19.93)	440 (15.55)
It is inconvenient for me to go get the vaccine	57 (17.59)	69 (22.92)	65 (22.49)	53 (17.79)	68 (18.23)	103 (35.52)	17 (5.54)	15 (4.32)	66 (21.93)	513 (18.13)
I cannot afford to pay for the vaccine	114 (35.19)	73 (24.25)	0 (0)	73 (24.5)	139 (37.27)	49 (16.9)	158 (51.47)	102 (29.39)	42 (13.95)	750 (26.50)
When the vaccine is available, I will not be prioritized as quickly as the wealthy/elite	148 (45.68)	173 (57.48)	48 (16.61)	5 (1.68)	88 (23.59)	146 (50.34)	2 (0.65)	0 (0)	92 (30.56)	702 (24.81)
I need my husband/family consent in order to get the vaccine	64 (19.75)	155 (51.5)	46 (15.92)	27 (9.06)	90 (24.13)	80 (27.59)	14 (4.56)	7 (2.02)	131 (43.52)	614 (21.70)
**Who influences your opinion about whether or not to take the COVID-19 vaccine?**										
Family or loved ones	134 (41.36)	143 (47.51)	80 (27.68)	109 (36.58)	228 (61.13)	114 (39.31)	55 (17.92)	21 (6.05)	151 (50.17)	1035 (36.57)
Religious leaders (imams, pastors, priests)	100 (30.86)	108 (35.88)	65 (22.49)	95 (31.88)	139 (37.27)	59 (20.34)	81 (26.38)	50 (14.41)	141 (46.84)	838 (29.61)
Community/tribal leaders	77 (23.77)	94 (31.23)	49 (16.96)	99 (33.22)	89 (23.86)	47 (16.21)	34 (11.07)	41 (11.82)	110 (36.54)	640 (22.61)
Political leaders	31 (9.57)	120 (39.87)	36 (12.46)	97 (32.55)	71 (19.03)	42 (14.48)	86 (28.01)	39 (11.24)	103 (34.22)	625 (22.08)
Celebrities/social media influencers	23 (7.10)	50 (16.61)	39 (13.49)	30 (10.07)	54 (14.48)	34 (11.72)	33 (10.75)	12 (3.46)	64 (21.26)	339 (11.98)
Healthcare workers	193 (59.57)	224 (74 .42)	124 (42.91)	122 (40.94)	212 (56.84)	172 (59.31)	92 (29.97)	174 (50.14)	207 (68.77)	1520 (53.71)
**Where do you get trusted and accurate information about the vaccine?**										
Television, radio, or newspaper	304 (93.83)	279 (92.69)	245 (84.78)	278 (93.29)	323 (86.6)	198 (68.28)	269 (87.62)	239 (68.88)	244 (81.06)	2379 (84.06)
Social media (Facebook, WhatsApp, Twitter, etc.)	69 (21.30)	97 (32.23)	61 (21.11)	20 (6.71)	189 (50.67)	117 (40.34)	72 (23.45)	68 (19.6)	87 (28.9)	780 (27.56)
Internet	73 (22.53)	99 (32.89)	62 (21.45)	23 (7.72)	184 (49.33)	153 (52.76)	64 (20.85)	44 (12.68)	112 (37.21)	814 (28.76)
Friends/family	195 (60.19)	204 (67.77)	147 (50.87)	193 (64.77)	265 (71.05)	160 (55.17)	135 (43.97)	152 (43.8)	180 (59.8)	1631 (57.63)
Religious bodies/leaders	204 (62.96)	191 (63.46)	142 (49.13)	199 (66.78)	208 (55.76)	117 (40.34)	180 (58.63)	105 (30.26)	194 (64.45)	1540 (54.42)
Medical professionals	274 (84.57)	272 (90.37)	236 (81.66)	276 (92.62)	335 (89.81)	241 (83.1)	227 (73.94)	206 (59.37)	264 (87.71)	2331 (82.37)
Government communications	275 (84.88)	248 (82.39)	251 (86.85)	266 (89.26)	308 (82.57)	191 (65.86)	264 (85.99)	108 (31.12)	244 (81.06)	2155 (76.15)
**When do you think a COVID-19 vaccine will be made available to you?**										
Already received the vaccine	11 (3.40)	4 (1.33)	64 (22.15)	19 (6.38)	102 (27.35)	81 (27.93)	40 (13.03)	10 (2.88)	13 (4.32)	344 (12.16)
Before the end of 2021	102 (31.448)	56 (18.6)	80 (27.68)	53 (17.79)	121 (32.44)	73 (25.17)	103 (33.55)	35 (10.09)	44 (14.62)	667 (23.57)
During 2022 (1st 6 months)	21 (6.48)	24 (7.97)	16 (5.54)	35 (11.74)	18 (4.83)	7 (2.41)	8 (2.61)	10 (2.88)	5 (1.66)	144 (5.09
During 2022 (2nd 6 months)	5 (1.54)	7 (2.33)	3 (1.04)	4 (1.34)	1 (0.27)	0 (0)	5 (1.63)	1 (0.29)	2 (0.66)	28 (0.99)
2023 or later	4 (1.23)	33 (10.96)	7 (2.42)	2 (0.67)	2 (0.54)	0 (0)	6 (1.95)	0 (0)	5 (1.66)	59 (2.08)
Never	28 (8.64)	77 (25.58)	77 (26.64)	1 (0.34)	39 (10.46)	20 (6.9)	93 (30.29)	240 (69.16)	23 (7.64)	598 (21.13)
Don’t know	153 (47.22)	99 (32.89)	42 (14.53)	183 (61.41)	90 (24.13)	106 (36.55)	52 (16.94)	46 (13.26)	209 (69.44)	982 (34.66)

The prevalence of vaccine hesitancy varied by country, but there was no consistent difference between participants living in rural and urban study areas or by age of the respondents (Table 4). Women were more likely to be vaccine hesitant than men (aPR 1.31, 95% CI: 1.19, 1.43). Respondents without education were more likely to be vaccine hesitant than those who had attended or completed primary school (aPR 1.13, 95% CI: 1.00, 1.28), but there was no difference among those with higher levels of education. Those who thought the vaccine was not very safe or not safe at all were more likely to be vaccine hesitant (aPR 1.70, 95% CI: 1.60,1.80). Similarly, people who thought the vaccine was not very effective or not effective at all were much more likely to be vaccine hesitant (aPR 3.02, 95% CI: 2.71,3.38). Those who reported they didn’t know if the vaccine was safe or effective were also more likely to be hesitant. A small proportion of participants (4%) said they did not trust traditional media, social media, friends, family, religious leaders, medical professionals, nor government communications for accurate information about the vaccine, and these respondents were more likely to be vaccine hesitant (aPR 1.25, 95% CI: 1.18,1.31). People who believed or were unsure about misinformation were much more likely to be vaccine hesitant (aPR 1.79, 95% CI: 1.52,2.11). People who thought the vaccine would be made available to them farther in the future (the next 1–2 years), never, or were unsure when it would become available were more vaccine hesitant (Table 4).

**Table 4:** Correlates of vaccines hesitancy (Respondent reporting that they would definitely not get it; maybe, but would wait and see what others do; or are undecided).

	N (%^1^) vaccine hesitant	Adjusted^2^ Prevalence Ratio (95% Confidence Interval)	P value
**Country**			
Burkina	177 (32.5)	1.19 (0.99,1.43)	0.06
Ethiopia	148 (28.8)	Reference	
Ghana	112 (41.9)	1.40 (1.15,1.71)	0.001
Nigeria	218 (33.7)	1.14 (0.96,1.36)	0.13
Tanzania	387 (64.6)	2.20 (1.89,2.56)	<0.001
**Rural Residence**	549 (38.5)	1.03 (0.94,1.12)	0.53
**Female sex**	546 (48.9)	1.31(1.19,1.43)	<0.001
**Age**			
20–29	166 (41.5)	1.10 (0.94,1.28)	0.24
30–39	265 (39.0)	Reference	
40–49	335 (42.1)	1.06 (0.92,1.21)	0.43
50+	275 (39.6)	1.07 (0.83,1.37)	0.61
**Education** (categories)			
None	208 (38.7)	1.13 (1.00,1.28)	0.05
Some or completed primary school	381 (44.8)	Reference	
Some or completed secondary school	217 (40.8)	1.04 (0.93,1.17)	0.48
Tertiary school or more	232 (37.5)	1.08 (0.94,1.25)	0.25
**Perceived safety of the COVID-19 vaccine**			
Very or somewhat safe	333 (21.2)	Reference	
Not very safe or not safe at all	237 (82.9)	1.70 (1.60,1.80)	<0.001
Do not know	472 (66.5)	1.51 (1.43,1.60)	<0.001
**Perceived efficacy of the COVID-19 vaccine**			
Very or somewhat effective	365 (22.8)	Reference	
Not very effective or not effective at all	256 (81.5)	3.03 (2.71,3.38)	<0.001
Do not know	421 (64.3)	2.62 (2.53,3.13)	<0.001
**Where do you get trusted and accurate information about the vaccine?**			
Television, radio, or newspaper	798 (36.7)	0.72 (0.66,0.78)	<0.001
Social media or Internet	339 (37.1)	0.89 (0.81,0.98)	0.02
Friends/family	506 (34.3)	0.79 (0.73,0.87)	<0.001
Religious bodies/leaders	456 (32.7)	0.72 (0.66,0.79)	<0.001
Medical professionals	754 (35.4)	0.69 (0.63,0.75)	<0.001
Government communications	657 (33.2)	0.61 (0.56,0.67)	<0.001
No trusted sources	95 (84.1)	1.25 (1.18,1.31)	<0.001
**Believing or being unsure about COVID-19 vaccine misinformation**	933 (43.9)	1.79 (1.52,2.11)	<0.001
**When do you think a COVID-19 vaccine will be made available to you** ^**3**^			
Before the end of 2021	113 (18.2)	Reference	
During 2022	47 (29.4)	1.67 (1.58,1.76)	<0.001
2023 or later	25 (44.6)	1.28 (1.17,1.40)	<0.001
Never	486 (89.5)	1.40 (1.24,1.59)	<0.001
Don’t know	371 (43.85)	1.38 (1.31,1.46)	<0.001

^1^ Among n = 2568, excluding n = 226 who did not answer the question.

^2^ Adjusted for country, rural area, respondent age, sex.

^3^ Among n = 2226 not yet vaccinated.

**Fig 2:**
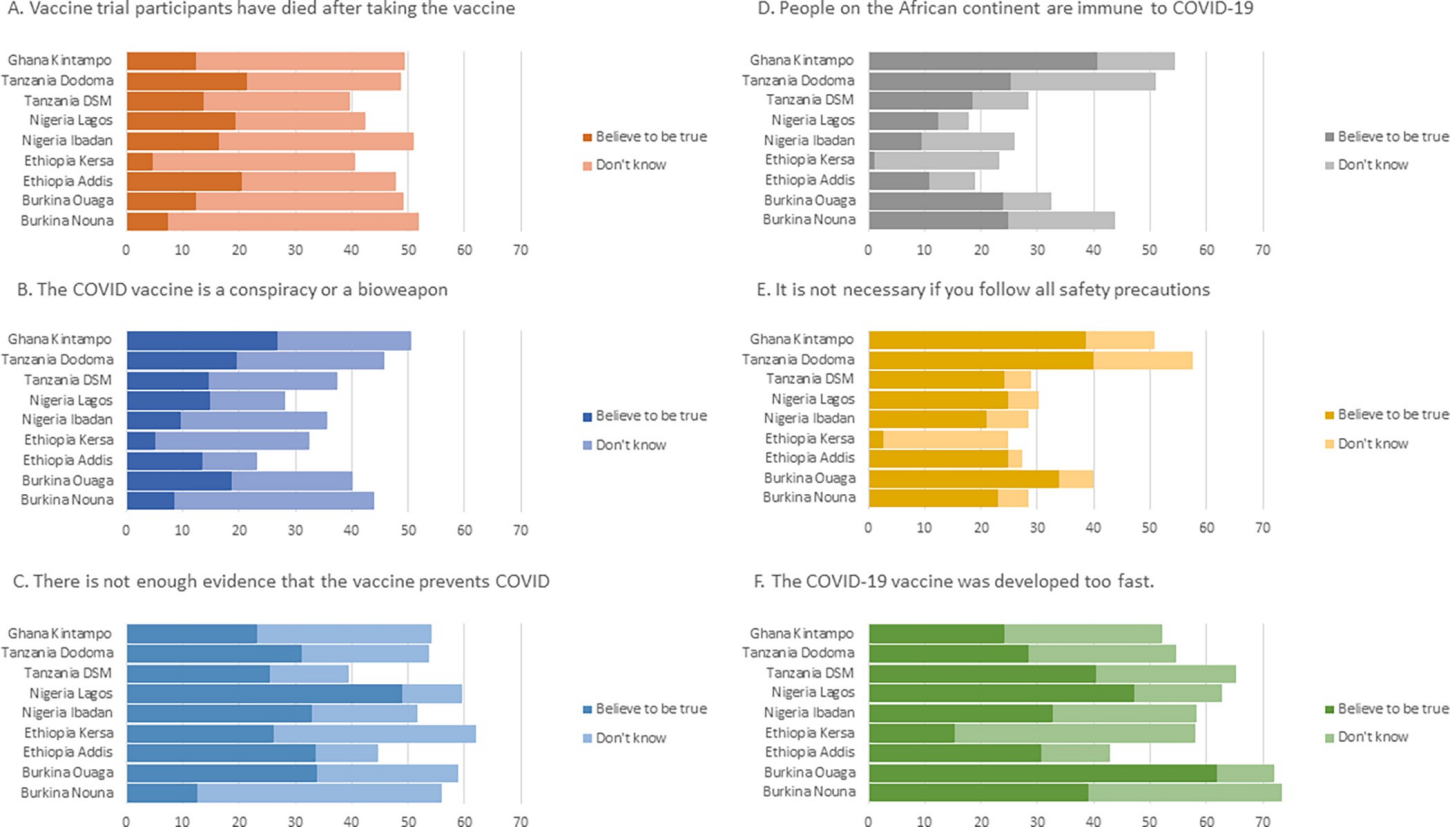
Proportion of respondents who believe or are unsure about vaccine falsehoods by study site.

Across all countries, 83% of participants believed at least one falsehood or point of misinformation about the COVID-19 vaccine and the likelihood of believing or being unsure about vaccine misinformation varied widely by country (Table 5). The most common misperception was that the vaccine was developed too fast where 35% of people thought it was true and 25% weren’t sure; this belief was most common in Burkina Faso and least common in Ethiopia (Fig 2). The second most common misperception was that there was not enough evidence that the vaccine prevents COVID-19. The least commonly believed falsehood was that people on the African continent are immune to COVD-19 (19% true; 15% unsure); however, the belief varied widely between study areas with 40% of respondents in Ghana and only 1% in Kersa stating this was true. Women were slightly more likely to believe at least one of these falsehoods (aPR 1.05, 95% CI 1.02, 1.08). People who said they did not have a source for trusted and accurate information about the vaccine were more likely to believe in at least one falsehood (aPR 1.09, 95% CI 1.02,1.16) (Table 5).

**Table 5:** Predictors of believing or being unsure about COVID-19 misinformation.

	N (%) Believe at least 1 piece of vaccine misinformation	Adjusted^1^ Prevalence Ratio (95% Confidence Interval)	P value
**Country**			
Burkina	578 (92.5)	1.2 (1.14,1.27)	<0.001
Ethiopia	454 (77.3)	Reference	
Ghana	273 (90.7)	1.18 (1.11,1.25)	<0.001
Nigeria	555 (83.7)	1.08 (1.02,1.14)	0.009
Tanzania	489 (74.8)	0.95 (0.9,1.02)	0.15
**Rural Residence**	1374 (83.6)	1.00 (0.96,1.03)	0.85
**Female sex**	1021 (82.9)	1.05 (1.02,1.08)	0.001
**Age**			
20–29	353 (82.5)	1.00 (0.94,1.06)	0.94
30–39	635 (83.1)	Reference	
40–49	714 (81.2)	0.95 (0.91,1.00)	0.04
50+	646 (85.2)	0.97 (0.90,1.05)	0.49
**Education** (categories)			
None	572 (87.7)	1.01 (0.97,1.05)	0.59
Some or completed primary school	783 (81.5)	0.98 (0.93,1.03)	0.47
Some or completed secondary school	454 (81.8)	0.97 (0.92,1.04)	0.41
Tertiary school or more	509 (81.1)	Reference	
**Where do you get trusted and accurate information about the vaccine?**			
Television, radio, or newspaper	1937 (81.4)	0.93 (0.9,0.97)	<0.001
Social media or Internet	779 (80.6)	0.95 (0.91,0.98)	0.002
Friends/family	1291 (79.2	0.91 (0.89,0.94)	<0.001
Religious bodies/leaders	1255 (81.5)	0.95 (0.92,0.98)	0.001
Medical professionals	1913 (82.1)	0.94 (0.91,0.97)	<0.001
Government communications	1782 (82.7)	0.96 (0.92,0.99)	0.007
No trusted sources	141 (94.6)	1.09 (1.02,1.16)	0.009

^1^ Adjusted for country, rural area, respondent age, sex.

## Discussion

This multi-country study investigated uptake behaviors of COVID-19 vaccines, focusing on risk perceptions and predictors of readiness to accept vaccines. Surveys for this study were conducted at a time when COVID-19 vaccination coverage was generally low; only 4 percent of the population in SSA were fully vaccinated during the study period [25]. For additional context, mobile phone penetration in SSA is high and continues to grow; it is expected that 84 percent of the population will have access to subscriber identity module card connection by 2025 [26]. The prevalence of vaccine hesitancy varied by country (Ethiopia 29%, Burkina Faso 33%, Nigeria 34%, Ghana 42%, Tanzania 65%), and within country, but not consistently by rural or urban context. Women were slightly more likely to be vaccine hesitant and to believe misinformation. While some trends were similar between study areas and countries, there was clear national and sub-national variation in the prevalence of beliefs surrounding COVID-19 vaccines. This study contributes to addressing substantial knowledge gaps around vaccine hesitancy discourse in Africa, where vaccine coverage is among the lowest in the world.

### Prevalence of vaccine hesitancy

A global survey conducted in June 2020 found that anticipated acceptance of a COVID-19 vaccine in many countries was far from universal [27]. Findings from surveys on intentions to accept the COVID-19 vaccines before and after vaccine introduction vary and continue to change over time and place across the globe [28]. Our study showed varying levels of willingness to accept COVID-19 vaccines across the five study countries. The proportion of respondents who reported readiness to receive a vaccine was highest in Ethiopia (62%) followed by Burkina Faso (58%) and Ghana (47%). In line with our study, a survey done across 15 countries in Africa revealed that willingness to get the COVID-19 vaccine was higher in Ethiopia than other countries [29]. In Tanzania, a considerable amount of vaccine hesitancy was reported by more than half of the respondents, compared with those willing to take up the COVID-19 vaccine (14%). Vaccine acceptance in our study was comparatively lower than those found in China, Ireland, and the UK [30,31]. A similar study conducted in late 2020 surveyed acceptance and hesitancy across thirteen lower-middle-income countries in Africa, Asia, and Latin America reported a higher acceptance rate, averaging 80% which contrasts significantly with our study [32]. The study included national surveys in Burkina Faso, Rwanda, and Sierra Leone and subnational surveys in Mozambique, Nigeria, and Uganda. The differences between our studies might reflect changing opinions towards the vaccine or national and sub-national differences in vaccine hesitancy across sub-Saharan Africa. The CDC survey report also noted a difference in willingness across surveyed countries.

The prevalence of vaccine hesitancy and belief in misinformation varies by social and demographic groups globally. Surveys in the US and UK found that people in older age groups, with lower educational attainment, and those who are unemployed are more likely to believe in COVID-19 vaccine-related misinformation [33]. Similarly, our study found that country of residence, being female or older, and preferred information sources were determinants of COVID-19 vaccine-related misinformation. Other studies have found that lower education levels, rural residence, and incomes have been associated with lower health literacy rates and lower uptake of vaccines [34,35], but these were not associated with believing or being unsure about COVID-19 vaccine-related misinformation in our study. This could be due to the fact that in the current study 42% of the participants have at least completed high school which aids in a better understanding of health information.

Sex and gender are important to consider in understanding the biology, social dynamics, and policy contexts related to infectious diseases [36]. We found that women are much more likely to be vaccine hesitant than men in our surveys, but the reasons for this gender difference is not clear. Though several women in the survey did raise concerns about their eligibility for or the safety of the vaccine during pregnancy or breastfeeding. As pregnant women were excluded from the initial COVID-19 vaccine clinical trials, the WHO recommended the vaccine only when the benefits outweigh the risks through the end of 2021 [37]. Many country programs have thus been slow to include pregnant and lactating women in national vaccine programs [38]. In fact, at the time of our survey, more than 100 countries did not explicitly recommend the vaccination of pregnant and lactating women [39]. Hesitancy among women may be compounded by other gendered barriers to vaccine access such as needing permission to get vaccinated or requiring support from family members for travel or childcare. National vaccine campaigns must consider gender in their design given the potential reasons for hesitancy and barriers to access. This is especially important given the majority of the global health workforce is female and thus deserves priority access to vaccines [40].

### Misinformation

Willingness to get the COVID-19 vaccines is influenced by different factors, and the spread of misinformation is one major problem that has been rampant in the context of the current pandemic mainly due to the many uncertainties [33,41]. Studies conducted in different settings revealed that people who believed that the COVID-19 vaccine was unsafe were less willing to receive the vaccine and more likely to believe COVID-19 vaccine misinformation [34]. However, in our study, while it was somewhat common to believe or be unsure about vaccine falsehoods, most people did not list these falsehoods as a reason they would not get the vaccine. Less than 5% of study respondents said that factors like fear of getting the illness, infertility, microchipping, and new world order were reasons they would not get the vaccine. Even though the proportion of people who cited these specific types of misinformation seems to be low, close to half of the participants believed that COVID-19 vaccines were not needed. The proportion was much higher in Dar es Salaam, Tanzania and in both sites in Burkina Faso. Respondents from Ghana were least likely to raise such concerns. Perhaps this is due to a substantial amount of misinformation circulating on social media, including those falsehoods not assessed in the current study [41]. Further, there have been many instances of political leaders or government officials sharing COVID-19-related misinformation. Delivering tailored, accurate and easily understandable COVID-19 vaccine-related information considering the variety of socioeconomic backgrounds would help the community to make informed decisions which in turn will improve the vaccine uptake. In addition, a better understanding of the sources of the misinformation and the myths will help to address the problem through designing tailored interventions [42].

A survey conducted by Africa CDC across 15 African countries reported that two-thirds of the respondents mentioned TV and radio as the most trusted sources for information for COVID-19 [29]. Similarly, the majority of our respondents mentioned that they received information on COVID-19 vaccines from mainstream media sources including TV, radio and newspapers which may have contributed to positively shaping respondents’ perception about vaccines. Earlier studies which showed that the proportion of hesitant respondents among receivers of information from institutional websites and mainstream media is lower than users of social media [43,44]. This is consistent with our findings that only 5% of respondents believes misinformation such as fear of getting illness or autism from the vaccine, concerns regarding infertility, fear of microchipping.

Previous studies have shown that non-official sources of health information influence decision making. Trust in such sources hinges on perceived motive and ability as well as whether they have been competent and reliable in the past. These non-official sources include family members and friends as well as advice from alternative health networks, politicians, celebrities and religious organizations. Consistent with other studies [32], we find the major reason for respondent readiness to accept vaccines were personal protection and family safety in all five study countries; however, marked differences existed with respect to the influence of family and doctors’ approval of vaccines. The influence of family was moderately high among respondents from Burkina Faso (67% urban; 56% rural) and Ghana (65%) as compared with Tanzania (11% urban; 31% rural) and Lagos, Nigeria (36%).

### Vaccine equity

Barriers to global vaccination efforts have left over 3 billion people unvaccinated or only partially vaccinated to date [45]. Vaccine hesitancy and anti-vaccine movements have slowed vaccination efforts, despite abundant evidence supporting the benefits of vaccination [46]. Unequal access to vaccines has prevented vaccination in low- and middle-income countries (LMICs), despite calls from WHO [47] and the UN Development Programme [48], to achieve equitable distribution and production of COVID vaccines [46]. Covid-19 vaccine availability differs tremendously across the globe. Several high-income countries have exceeded 90% coverage, but only about 11% in low-income countries have received at least one dose [49]. By December 30, 2021, only seven African countries had achieved their targeted 40% vaccination rates [50]. It is essential to achieve vaccine equity alongside public health campaigns to deal with misconceptions as well as fundamental issues including health infrastructure, trained personnel, medical equipment, unreliable supply and distribution of vaccines. Equally crucial for vaccine equity is empowering LMICs to develop and grow their own vaccine manufacturing capabilities to provide a long-term and sustainable solution to accomplishing global vaccination coverage, for COVID-19 and many other communicable diseases [46]. Respondents who thought the vaccine would be made available to them farther in the future (the next 1–2 years), never, or were unsure when it would become available were more vaccine hesitant. This could be considered another challenge related to vaccine equity.

### Strengths & limitations

This study has major strengths including the inclusion of urban and rural areas in five countries in sub-Saharan Africa which allowed us to assess vaccine hesitancy among diverse settings. In addition, uniformity of tools and data collection approach allowed comparability of findings across the nine study areas. CATI is known to generate data that is comparable to a face-to-face interview when compared with other methods of phone surveys [51]. There are few limitations of this study, the first of which is linked with the data collection approach. Households that own telephones (landline or mobile) were included in the survey which limits the generalizability of the findings as those without phones were not reached. Additionally, participants were not selected to be representative of the larger study areas and countries, which limits the generalizability of these findings. Lastly, self-reported data about hesitancy is not the same as the actual behavior and practices of the participants related to the COVID-19 vaccine. More recent data from national health systems shows increasing vaccine coverage even in countries where we found hesitancy to be quite high.

### Conclusions

Ensuring reliable global access to COVID-19 vaccines and addressing the underlying causes of vaccine hesitancy will be key in combating the COVID-19 pandemic and promoting good health in the post-pandemic period. Educational campaigns targeted at misinformation and tailored to suit each country at both national and sub-national levels are recommended to build trust in COVID-19 vaccines and reduce hesitancy.

## Supporting information

S1 Checklist Inclusivity in global health. (DOCX)

S1 Fig Flow chart of sampled households and complete interviews, by country. (TIF)
