# Supplementary figures and images for: Determinants of COVID-19 vaccine readiness and hesitancy among adults in sub-Saharan Africa
Source: PLOS Glob Public Health. 2023 Jul 14;3(7):e0000713. doi: 10.1371/journal.pgph.0000713 (PMC10348558; doi:10.1371/journal.pgph.0000713)

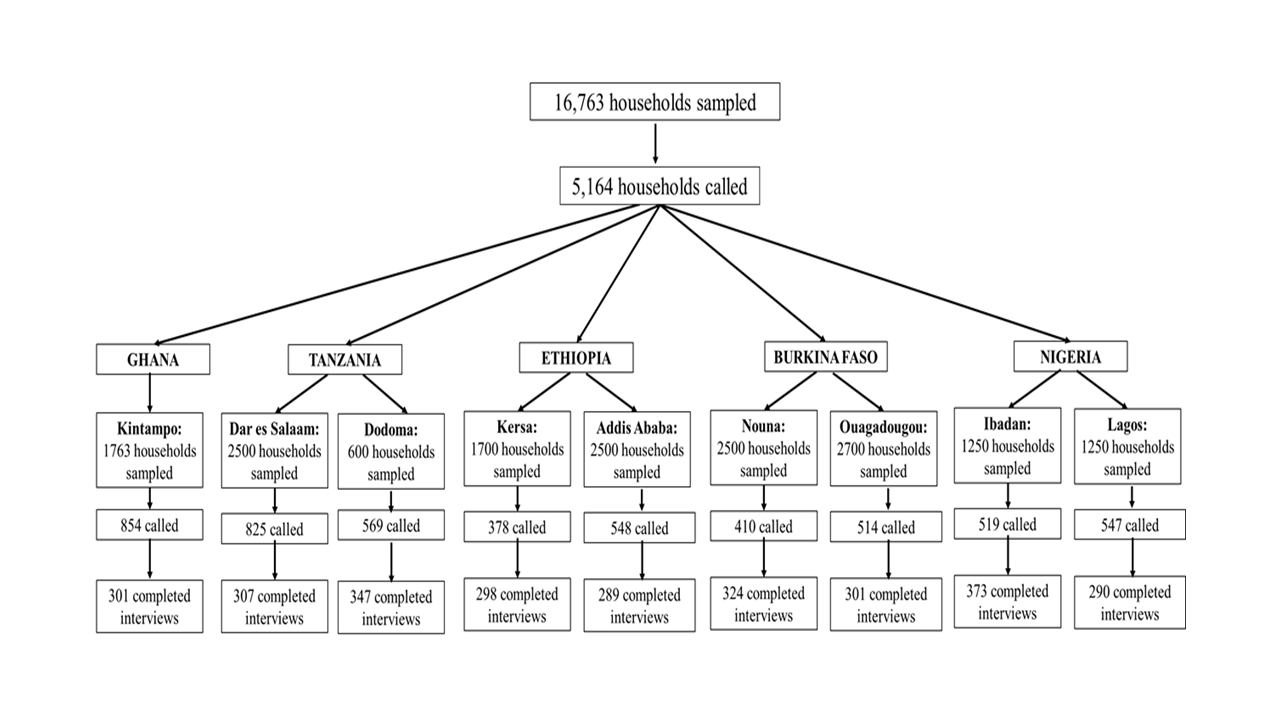

Supplement: S1 Fig — (TIF) [file pgph.0000713.s002.tif]
